# *In silico* analyses reveal common cellular pathways affected by loss of heterozygosity (LOH) events in the lymphomagenesis of Non-Hodgkin’s lymphoma (NHL)

**DOI:** 10.1186/1471-2164-15-390

**Published:** 2014-05-21

**Authors:** Carlos Aya-Bonilla, Emily Camilleri, Larisa M Haupt, Rod Lea, Maher K Gandhi, Lyn R Griffiths

**Affiliations:** Genomics Research Centre, Institute of Health and Biomedical Innovation, Queensland University of Technology, Brisbane, Australia; Department of Orthopedic Surgery, Mayo Clinic, Rochester, MN USA; Department of Haematology, Princess Alexandra Hospital, Brisbane, Australia; Centre for Experimental Haematology, Translational Research Institute, Brisbane, Australia

**Keywords:** LOH, PTPRJ, Interactome, Pathway analysis, NHL

## Abstract

**Background:**

The analysis of cellular networks and pathways involved in oncogenesis has increased our knowledge about the pathogenic mechanisms that underlie tumour biology and has unmasked new molecular targets that may lead to the design of better anti-cancer therapies. Recently, using a high resolution loss of heterozygosity (LOH) analysis, we identified a number of potential tumour suppressor genes (TSGs) within common LOH regions across cases suffering from two of the most common forms of Non-Hodgkin’s lymphoma (NHL), Follicular Lymphoma (FL) and Diffuse Large B-cell Lymphoma (DLBCL). From these studies LOH of the protein tyrosine phosphatase receptor type J (*PTPRJ*) gene was identified as a common event in the lymphomagenesis of these B-cell lymphomas. The present study aimed to determine the cellular pathways affected by the inactivation of these TSGs including *PTPRJ* in FL and DLBCL tumourigenesis.

**Results:**

Pathway analytical approaches identified that candidate TSGs located within common LOH regions participate within cellular pathways, which may play a crucial role in FL and DLBCL lymphomagenesis (i.e., metabolic pathways). These analyses also identified genes within the interactome of PTPRJ (i.e. PTPN11 and B2M) that when inactivated in NHL may play an important role in tumourigenesis. We also detected genes that are differentially expressed in cases with and without LOH of *PTPRJ*, such as *NFATC3* (nuclear factor of activated T-cells, cytoplasmic, calcineurin-dependent 3). Moreover, upregulation of the VEGF, MAPK and ERBB signalling pathways was also observed in NHL cases with LOH of *PTPRJ*, indicating that LOH-driving events causing inactivation of *PTPR*J, apart from possibly inducing a constitutive activation of these pathways by reduction or abrogation of its dephosphorylation activity, may also induce upregulation of these pathways when inactivated. This finding implicates these pathways in the lymphomagenesis and progression of FL and DLBCL.

**Conclusions:**

The evidence obtained in this research supports findings suggesting that FL and DLBCL share common pathogenic mechanisms. Also, it indicates that PTPRJ can play a crucial role in the pathogenesis of these B-cell tumours and suggests that activation of PTPRJ might be an interesting novel chemotherapeutic target for the treatment of these B-cell tumours.

**Electronic supplementary material:**

The online version of this article (doi: 10.1186/1471-2164-15-390) contains supplementary material, which is available to authorized users.

## Background

The identification of altered pathways in tumour cells has provided a more holistic understanding of the pathogenic mechanisms that underlie the genesis, progression and chemoresponse of cancer. As a consequence, malfunction of a gene or a group of genes must be analysed as part of a complex network of components that are highly related to each other. However, this analysis is limited by factors such as the lack of analytic tools to convert and integrate, in a feasible and reliable manner, large amounts of data derived from different high-throughput genomic approaches into outputs with more biological meaning.

Non-Hodgkin’s lymphoma (NHL) represents a highly biological and clinical heterogeneous group of blood cancers [1, 2]. Two of the most common NHL subtypes are Diffuse Large B-cell Lymphoma (DLBCL), an aggressive lymphoma, and Follicular lymphoma (FL), a slow-growing type of lymphoma, which account for around 50% of NHL cases [1, 3]. The poor treatment outcomes obtained between 20-40% of cases with these NHL lymphomas has prompted studies aimed at the discovery of genes and pathways that could act as novel targets for a therapy that increase the survival rates of patients suffering from NHL [4–6]. However, the high genetic variability observed within NHL subtypes, has limited the understanding of the pathology of these NHL subtypes, as well as the discovery of new molecular targets for therapeutic development.

In a previous study, the integration of copy number (CNV) and gene expression profiling (GEP) data from DLBCL and FL cases, allowed us to identify common and disease-specific genetic alterations targeting known oncogenic pathways, such as the mitogen activated protein kinase (MAPK) and apoptosis signalling pathways, unmasking common pathogenic mechanisms underlying the malignant phenotype of these biologically and genetically distinct NHL subtypes [7]. Likewise, in a recent study, using a high resolution loss of heterozygosity (LOH) analysis in FLs and DLBCLs, we also identified candidate tumour suppressor genes (TSGs) within common LOH regions across these NHL subtypes and implicated them in the lymphomagenesis of these B-cell lymphomas [8]. In this study, we have performed pathway analysis of these candidate TSGs, in order to identify common cellular networks that might be altered by the inactivation of one or more TSGs in the lymphomagenesis of these B-cell lymphomas.

As part of our recent LOH studies, we also implicated *PTPRJ* (protein tyrosine phosphatase receptor type J) as a novel TSG in the tumourigenesis of FL and DLBCL, with LOH of *PTPRJ* identified as a common event in FL and DLBCL. LOH of PTPRJ was also confirmed by a decrease of heterozygosity of a microsatellite targeting *PTPRJ* loci in these NHL cases. In addition, FL cases with LOH exhibited a significant downregulation of *PTPRJ* [8]. Several lines of evidence support the tumour suppressive role of *PTPRJ*, as this protein tyrosine phosphatase has been implicated in the oncogenesis of breast, lung, colorectal, thyroid and meningioma cancers [9–12]. Furthermore, PTPRJ regulates signalling pathways involved in cell growth, proliferation and angiogenesis, such as MAPK (ERK1/2), PLCG1, PI3K (p85), FLT3, B-cell receptor (BCR), PDGFRB and VEGFR2 signalling [13–19]. Nonetheless, the natural ligands of PTPRJ, Syndecan-2 (SDC2), a transmembrane heparan sulfate proteoglycan, and Thrombospondin-1 (THBS1), a homotrimeric glycoprotein, have been identified to induce cell adhesion and inhibit cell growth and angiogenesis, respectively [20, 21]. This anti-tumour activity of PTPRJ has also been demonstrated in *In vitro* experiments, using agonist peptides of PTPRJ and the oncogenic silencing of PTPRJ expression by microRNA-328 expression [22–24].

Since the role of PTPRJ in normal and malignant B-cell differentiation is poorly understood, we used pathway and genomic analyses to identify cellular pathways that may be altered by the inactivation of *PTPRJ* in order to provide a better understanding of the role of PTPRJ in the lymphomagenesis of FL and DLBCL. These analyses identified metabolic pathways as one of the most enriched and affected pathways resulting from the inactivation of candidate TSGs, indicating that these cellular pathways might play an important role in FL and DLBCL tumourigenesis. Additionally, inactivation of *PTPRJ* was shown to affect the expression of a number of genes and pathways that are regulated by PTPRJ through protein-protein interactions.

## Results

### Candidate tumour suppressor genes (TSGs) targeted by LOH events participate within common cellular networks that may orchestrate NHL lymphomagenesis

**Figure 1:**
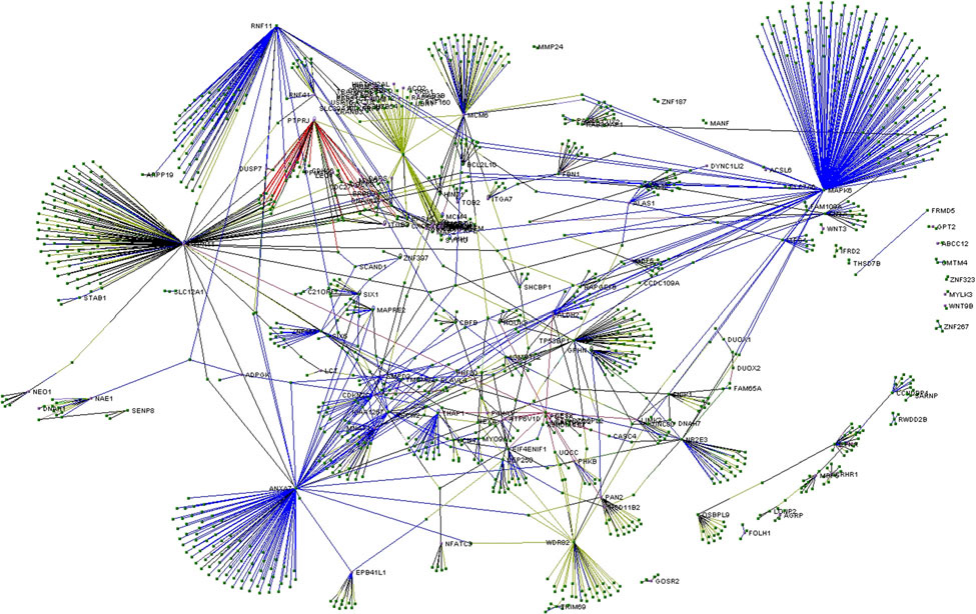
**Global interactome of genes commonly affected by LOH across NHL patients. An initial list containing 262 genes was used; however, only those with more than one interaction are shown.** A total of 1270 nodes belonging to 68 pathways were mapped, which indicates the high level of interaction among the genes targeted by LOH-driving events in NHL tumours. The genes with labels correspond to the questioned LOH genes.

In order to determine whether the candidate tumour suppressor genes (TSGs) located within common LOH regions across DLBCL and FL cases interact and participate within common cellular networks, a global interactome of a total of 262 genes affected by LOH events [8], was created using the VisANT (v. 4.06) platform (Figure 1). As a result of this analysis, the METABOLIC pathway (KEGG hsa-01100) was identified as the most enriched pathway by these candidate TSGs. This approach also revealed a high level of interaction amongst these candidate TSGs targeted by LOH events, indicating that NHL tumourigenesis might be orchestrated by the possible inactivation of these candidate TSGs, which could lead to the constitutive activation of oncogenic pathways. In this context, it is important to highlight that the genes *MAPK6* (mitogen-activated protein kinase 6), *PTPN11* (protein tyrosine phosphatase, non-receptor type 11) and *ANXA7* (annexin A7) showed the highest number of links with other genes (170, 122 and 106 respectively). The high number of protein links of these proteins suggests that a possible inactivation of these genes may cause a major deregulation of patwhays by altering the function of a high number of genes. Thus, these genes are shown as the most interesting candidate TSGs in NHL lymphomagenesis.

In addition to the global interactome study, a gene set enrichment analysis (GSEA) was performed to classify the 262 candidate TSGs targeted by LOH events, thenceforth also referred in this study as LOH genes, into curated gene families and also to determine the pathways that are mostly enriched with these genes. This approach categorized these LOH genes into gene families (Additional file 1). For instance, *FASLG* was classified into the cytokines and growth factor family and together with *PTPRJ* into the cell differentiation markers family. *EP300* was found in the translocated cancer gene, transcription factor and tumour suppressor gene families. Additionally, *MAPK6, PTPN11* and *NFATC3* were identified as members of the protein kinases, oncogenes and transcription factors families, respectively.

Furthermore, 215 out of 262 genes within LOH regions were identified and contrasted with collections from the molecular signature database (MsigDB, v3.1). Several cellular pathways were identified to be enriched with genes located within LOH regions (Table 1). The most enriched pathways for LOH genes were the, KEGG_ARGININE_AND_PROLINE METABOLISM, REACTOME_DOUBLE STRAND_BREAK_REPAIR and MICROTUBULE_ASSOCIATED_COMPLEX GO (gene ontology) gene sets. Interestingly, the TSGs *TP53BP1* and *B2M* were identified as members of the REACTOME_DOUBLE_STRAND_BREAK_REPAIR and BIOCARTA_CTL PATHWAY pathways, respectively.

**Table 1:** **List of cellular pathways enriched with genes within LOH regions**

Gene set name	K	Description	k	k/K*	***P*** value
KEGG_Arginine_and_proline_metabolism	54	Arginine and proline metabolism	6	0.1111	4.84 × 10^-4^
Reactome_double_strand_break_repair	24	Genes involved in Double-Strand Break Repair	3	0.125	9.73 × 10^-3^
Microtubule_associated_complex	47	Genes annotated by the GO term GO:0005875. Any multimeric complex connected to a microtubule.	4	0.0851	1.13 × 10^-2^
PID_S1P_S1P3_pathway	29	S1P3 pathway	3	0.1034	1.64 × 10^-2^
Reactome_unwinding_of_DNA	11	Genes involved in Unwinding of DNA	2	0.1818	1.71 × 10^-2^
Biocarta_FAS_pathway	30	FAS signaling pathway (CD95)	3	0.1	1.80 × 10^-2^
Reactome_downregulation_of_ERBB2_ERBB3_SIGNALING	12	Genes involved in Downregulation of ERBB2:ERBB3 signaling	2	0.1667	2.03 × 10^-2^
KEGG_Non_homologous_end_joining	14	Non-homologous end-joining	2	0.1429	2.73 × 10^-2^
PID_IL5_pathway	14	IL5-mediated signaling events	2	0.1429	2.73 × 10^-2^
Protein_kinase_binding	62	Genes annotated by the GO term GO:0019901. Interacting selectively with a protein kinase, any enzyme that catalyzes the transfer of a phosphate group, usually from ATP, to a protein substrate.	4	0.0645	2.84 × 10^-2^
Biocarta_CTL_pathway	15	CTL mediated immune response against target cells	2	0.1333	3.11 × 10^-2^
MIPS_Emerin_complex_24	15	Emerin complex 24	2	0.1333	3.11 × 10^-2^
Cytoskeletal_part	235	Genes annotated by the GO term GO:0044430. Any constituent part of the cytoskeleton.	9	0.0383	3.23 × 10^-2^
KEGG_Cell_cycle	128	Cell cycle	6	0.0469	3.28 × 10^-2^
Myosin_complex	16	Genes annotated by the GO term GO:0016459. A protein complex that functions as a molecular motor; uses the energy of ATP hydrolysis to move actin filaments.	2	0.125	3.51 × 10^-2^
PID_DNAPK_pathway	16	DNA-PK pathway in nonhomologous end joining	2	0.125	3.51 × 10^-2^
Kinase_binding	70	Genes annotated by the GO term GO:0019900. Interacting selectively with a kinase.	4	0.0571	4.17 × 10^-2^
Biocarta_MCM_pathway	18	CDK Regulation of DNA Replication	2	0.1111	4.36 × 10^-2^
Lipoprotein_binding	18	Genes annotated by the GO term GO:0008034.	2	0.1111	4.36 × 10^-2^

*k/K is the ratio between the number of genes in overlap (k) and the number of genes in gene set (K). This table combines the results from the collection of gene sets from canonical pathways, BioCarta, KEGG, Reactome, and GO (gene ontology) gene sets.

Significant enrichment of LOH genes was also observed in a collection of gene sets with chemical and genetic alterations (CGP), indicating that these candidate TSGs have been previously implicated in multiple forms of cancer (Additional file 2). In this collection, MARSON_BOUND_BY_FOXP3_STIMULATED, DIAZ_CHRONIC MEYLOGENOUS_LEUKEMIA_UP and GRAESSMANN_RESPONSE_TO_MC AND _DOXORUBICIN_UP were the one of most significant gene sets. To highlight, *PTPRJ* was included in the ROYLANCE_BREAST_CANCER_16Q_COPY_NUMBER_DN and *TP53BP1* was listed in the PUJANA_BREAST_CANCER_LIT_INT_NETWORK gene sets.

### Interactome of PTPRJ overlaps with the interactome of PTPN11 (protein tyrosine phosphatase, non-receptor type 11), a candidate TSG also found to be targeted by common LOH events in NHL

In a recent study, the protein tyrosine phosphatase receptor type J (*PTPRJ*) gene was implicated as a novel TSG in the lymphomagenesis of DLBCL and FL [8]. In this previous study, using publically available gene expression profiling data from our cohort of NHL cases and subsequent validation by qualitative PCR (qPCR), a significant downregulation of PTPRJ expression in FL cases with LOH, indicating that the LOH-driving events targeting *PTPRJ* might have an effect on mechanisms regulating the expression of *PTPRJ* [7, 8]*.* This significant reduction in PTPRJ transcript abundance may result in haploinsufficiency of this TSG and thus, play a crucial role in the FL and DLBCL tumourigenesis. Based on this, we performed *in silico* pathway and gene expression analyses in order to provide more information about the unclear role and function of PTPRJ in normal and malignant B-cell development and to understand the effect of LOH of *PTPRJ* in the lymphomagenesis of FL and DLBCL from a more cellular perspective.

**Figure 2:**
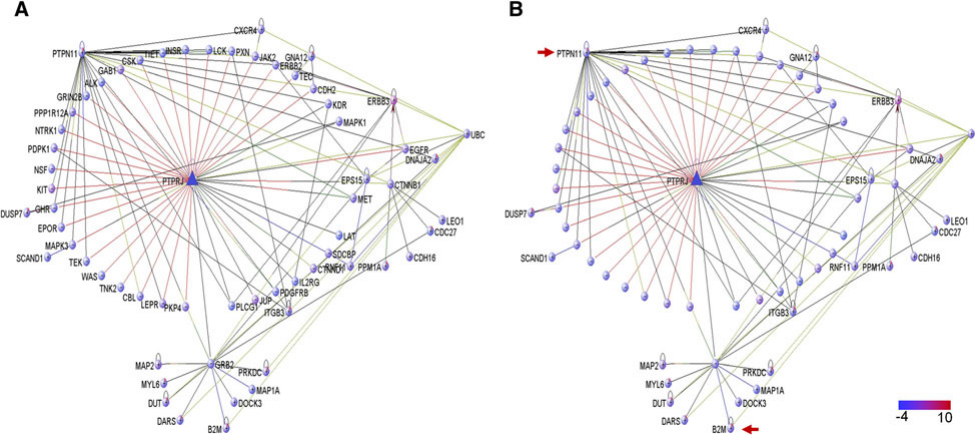
**Interactome of PTPRJ indicates that the inactivation of** ***PTPRJ*** **may affect cellular networks and that these networks are not only altered by inactivation of** ***PTPRJ*** **.** PTPRJ gene network showing **A)** all the known genes that interact with PTPRJ and **B)** only those genes that were also found to be inactivated in NHL cases. Two levels of interaction were used to filter the interactions. Interestingly, we found that the gene *PTPN11*, which was also found inactivated in NHL cases, shares some target genes with PTPRJ; moreover, B2M has an indirect interaction with PTPRJ (red arrows).

Initially, we studied the interactome of PTPRJ to determine whether candidate TSGs, affected by LOH events, interact directly or indirectly with PTPRJ and play a role together with PTPRJ, within common cellular networks. The analysis of the gene-gene interactions of PTPRJ revealed that none of the 41 genes, which are known to interact with PTPRJ, were previously identified among the genes located within the common LOH regions across FLs and DLBCLs (Figure 2) [8]. However, 22 genes that interact indirectly (one level) with PTPRJ were found to be commonly inactivated in NHL cases. Among these candidate TSGs, B2M (Beta-2_microglobulin) and PTPN11 (protein tyrosine phosphatase, non-receptor type 11) were highlighted. B2M was found to interact indirectly with PTPRJ, through the PTPRJ-GRB2-B2M interaction. PTPN11, another protein tyrosine phosphatase (PTP), was identified to share 24 interacting genes with PTPRJ. Additionally, using previously published high resolution LOH data, LOH of *PTPN11* was identified in 74% of NHL, 71% of FL and 76% of DLBCL cases [8].

The genes showing interaction with both *PTPRJ* and *PTPN11* are: *MAPK3* (mitogen-activated protein kinase 3), *GRB2* (growth factor receptor-bound protein 2), *ERBB2* (v-erb-b2 erythroblastic leukemia viral oncogene homolog 2), *MET* (met proto-oncogene (hepatocyte growth factor receptor), *PDGFRB* (platelet-derived growth factor receptor, beta polypeptide), *CTNNB1* (catenin (cadherin-associated protein), beta 1, 88 kDa), *PLCG1* (phospholipase C, gamma 1), *CBL* (Cas-Br-M (murine) ecotropic retroviral transforming sequence), *KIT* (v-kit Hardy-Zuckerman 4 feline sarcoma viral oncogene homolog), *NTRK1* (neurotrophic tyrosine kinase, receptor, type 1), *KDR* (kinase insert domain receptor (a type III receptor tyrosine kinase)), *PXN* (paxillin), *GAB1* (GRB2-associated binding protein 1), *LEPR* (leptin receptor), *EPOR* (erythropoietin receptor), *GHR* (growth hormone receptor), *CXCR4* (chemokine (C-X-C motif) receptor 4), *CSK* (c-src tyrosine kinase), *INSR* (insulin receptor), *JAK2* (Janus kinase 2), *LCK* (lymphocyte-specific protein tyrosine kinase), *GRIN2B* (glutamate receptor, ionotropic, N-methyl D-aspartate 2B), *TEK* (TEK tyrosine kinase, endothelial) and *TIE1* (tyrosine kinase with immunoglobulin-like and EGF-like domains 1). Interestingly, these 24 genes were found to significantly enrich important signalling pathways in lymphocyte biology, which suggests that a double inactivation of PTPRJ and PTPN11 might have an aberrant effect on the function of these pathways (Table 2).

**Table 2:** **List of gene sets commonly regulated by PTPRJ and PTPN11 based on gene expression**

Gene set name	K	k	k/K*	***P*** value
KEGG_Pathways_in_cancer	328	10	0.0305	6.86 × 10^-7^
KEGG_Focal_adhesion	201	8	0.0398	1.70 × 10^-6^
KEGG_ERBB_Signaling_pathway	87	6	0.069	1.81 × 10^-6^
KEGG_Cytokine_cytokine_receptor_interaction	267	8	0.03	1.42 × 10^-5^
KEGG_Neurotrophin_signaling_pathway	126	6	0.0476	1.57 × 10^-5^
KEGG_Adherens_junction	75	5	0.0667	1.76 × 10^-5^
KEGG_Prostate_cancer	89	5	0.0562	4.06 × 10^-5^
KEGG_JAK_Stat_signaling_pathway	155	6	0.0387	5.11 × 10^-5^
KEGG_Endometrial_cancer	52	4	0.0769	7.76 × 10^-5^
KEGG_Non_small_cell_lung_cancer	54	4	0.0741	9.01 × 10^-5^
KEGG_T_Cell_receptor_signaling_pathway	108	5	0.0463	1.03 × 10^-4^
KEGG_Endocytosis	183	6	0.0328	1.29 × 10^-4^
KEGG_Chemokine_signaling_pathway	190	6	0.0316	1.59 × 10^-4^
KEGG_Glioma	65	4	0.0615	1.87 × 10^-4^
KEGG_Renal_cell_carcinoma	70	4	0.0571	2.49 × 10^-4^
KEGG_Thyroid_cancer	29	3	0.1034	2.81 × 10^-4^
KEGG_VEGF_signaling_pathway	76	4	0.0526	3.42 × 10^-4^
KEGG_Leukocyte_transendothelial_migration	118	4	0.0339	1.80 × 10^-4^
KEGG_Acute_myeloid_leukemia	60	3	0.05	2.40 × 10^-3^

*k/K is the ratio between the number of genes in overlap (k) and the number of genes in gene set (K).

### LOH of *PTPRJ* induces deregulation of genes and signalling pathways in NHL tumours

**Figure 3:**
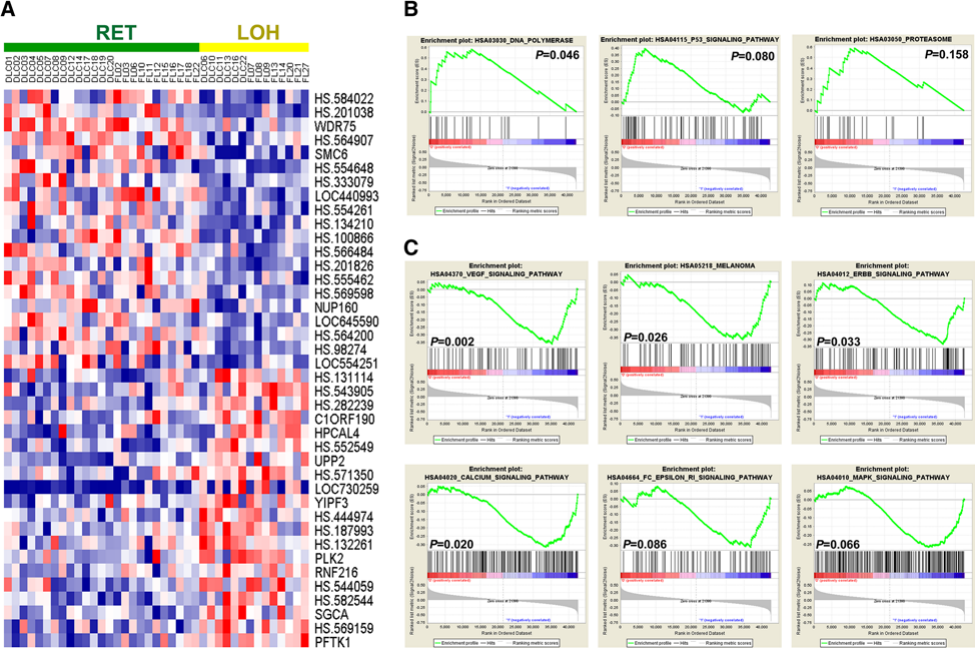
**Genes and pathways affected by LOH of** ***PTPRJ*** **. A)** Heatmap of genes differentially expressed between cases with retention (RET) and LOH calls. **B)** DNA polymerase (HSA03030), TP53 signaling pathway (HSA04115) and Proteasome (HSA03050) were identified as the most enriched gene sets in cases with retention of *PTPRJ*. **C)** In cases with LOH, the gene sets VEGF signaling pathway (HSA04370), Melanoma (HSA05218), ERBB signaling pathway (HSA04012), Calcium signaling pathway (HSA04020), FC Epsilon RI signaling pathway (HSA04664) and MAPK signaling pathway (HSA04010) were the most enriched. Despite an FDR (false discovery rate) of 1 for all the enrichments, the results from this gene-set enrichment analysis were analysed based on the P values instead of the FDR scores. The high FDR values obtained in this analysis may be due to the low population size. The lists of all gene sets from this analysis are provided in Additional file 3 and Additional file 4.

We also used gene expression profiling (GEP) data from NHL patients with known LOH status for *PTPRJ* [7], to detect genes that are significantly deregulated by LOH of *PTPRJ.* Using this approach, we identified differentially expressed genes between cases with and without LOH of *PTPRJ*. This result indicates that LOH of *PTPRJ* could have an effect on the expression pattern of some genes (Figure 3A), suggesting that some pathways might be differentially enriched between the two categories. In order to prove this hypothesis, a Gene Set Enrichment Analysis (GSEA) identified some cellular pathways with significant upregulation in cases with retention and some others, in cases with LOH (Figure 3B and 3C). The genes that were upregulated in the most significant gene sets are described in Table 3. The lists of the gene sets enriched in each LOH status (RET or LOH) are detailed in the Additional file 3 and Additional file 4. Nonetheless, it is important to highlight that despite all enrichment analyses exhibiting FDR values equal to 1, the results from these gene-set enrichments, described in Figure 3 and Table 3, were analysed based on the nominal significance (P value lower than 0.05) instead of the FDR values, due to the low population size, which could be driving the high FDR scores, and the high biological relevance of the gene-sets that were significantly upregulated in cases with LOH of *PTPRJ*.

**Table 3:** **List of genes enriched within the gene sets differentially expressed between cases with retention and LOH of PTPRJ**

Gene sets	Genes
Upregulated in RET cases	
HSA03030_DNA_Polymerase	*POLQ,PRIM1,POLD3,POLE3,POLK,POLE2,POLE4,POLS,REV1,POLA2,RFC5,POLD1,POLB,POLI,POLE*
HSA04115_P53_Signaling_ pathway	*CDC2,PTEN,TNFRSF10B,CHEK1,PMAIP1,CDK4,CDK2,SFN,PERP,P53AIP1,FAS,CCNB1,SERPINB5,CASP8,CCNB2,EI24,CCND2,TP53I3,CCNE2,RFWD2,CCNB3,MDM2*
HSA03050_Proteasome	*PSMB4,PSMA3,PSMA4,PSMA1,PSMB7,PSMD11,PSMD13,PSMD1,PSMC3,PSMA2,PSMA5,PSMD2,PSMA7,PSMA6*
Upregulated in LOH cases	
HSA04370_VEGF_signaling_pathway	*MAPKAPK2,PLA2G10,PXN,PLA2G6,PLA2G2F,MAP2K1,MAP2K2,RAF1,PRKCG,PIK3R1,PLA2G1B,MAPK3,MAPK12,SPHK2,AKT3,PLA2G2A,BAD,RAC1,PLA2G5,PIK3CD,NFATC3,PRKCA,PIK3CG*
HSA05218_Melanoma	*FGF9,CCND1,MET,FGF17,FGF22,MAP2K1,MAP2K2,IGF1,RAF1,PIK3R1,MAPK3,CDK6,AKT3,FGF8,BAD,FGF23,ARAF,FGF12,FGFR1,PIK3CD,PDGFRA,PDGFD,FGF7,PIK3CG*
HSA04012_ERBB_signaling_pathway	*CAMK2G,SHC4,MAP2K1,MAP2K2,RAF1,STAT5B,PRKCG,PAK2,NRG3,RPS6KB2,CAMK2B,PIK3R1,PAK4,NRG1,MAPK3,ERBB2,AKT3,TGFA,BAD,ARAF,CBL,ERBB3,PIK3CD,PRKCA,PIK3CG, PAK3*
HSA04020_Calcium_ signaling_pathway	*GNA11,HTR4,CCKBR,CHP,ADRA1D,ITPR1,RYR2,SLC25A5,PHKA1,NOS1,PHKA2,SLC8A2,CACNA1C,ATP2B1,MYLK,AGTR1,CAMK2G,CHRNA7,CAMK4,MYLK2,PRKCG,CAMK2B,GRM5,BDKRB1,ADCY8,RYR1,ERBB2,SPHK2,TRHR,CALM3,PRKACG,ADORA2A,PLN,GNA14,TBXA2R,PTAFR,ERBB3,ADCY9,P2RXL1,TACR2,CACNA1D,PDGFRA,HRH1,NTSR1,PLCB4,HTR2B,PRKCA, CACNA1I,SLC25A6,PTGER3,ITPKB*
HSA04664_FC_epsilon_RI_ signaling_pathway	*PLA2G2F,VAV3,MAP2K1,MAP2K2,RAF1,PIK3R1,PLA2G1B,MAPK3,MAPK12,AKT3,PLA2G2A,RAC1,IL4,PLA2G5,PIK3CD,IL5, PDK1,PRKCA,PIK3CG*
HSA04010_MAPK_signaling_pathway	*DUSP8,MAP2K7,FGF21,CACNA2D3,MEF2C,FGF5,RPS6KA3,CACNA2D1,TGFBR2,TAOK2,CACNA2D2,RAC3,NFKB2,CHP,FLNA,SRF,FGF9,DAXX,RPS6KA6,RRAS,MAPKAPK2,PLA2G10,NTRK1,CACNA1C,PLA2G6,FGF17,FGF22,RAP1A,FLNC,MAP3K14,PLA2G2F,CACNB4,MAP2K1,GADD45A,RPS6KA1,NF1,MAP2K2,RPS6KA5,RAF1,PRKCG,PAK2,TAOK3,MOS,MAPK8IP3,PLA2G1B, MAPK3,MAPK12,RRAS2,PRKACG,GADD45G,AKT3,NTF5,FGF8, PLA2G2A,FLNB,RAC1,FGF23,FGF12,RASGRF2,FGFR1,PLA2G5,MAP3K8NLK PTPN5 CACNA1D IL1R1 PDGFRA ARRB2 MAP3K10 RPS6KA4 PPP5C FGF7 GNA12 CACNG5 MAP3K13 DUSP7 PRKCA MAP2K5 CACNA1I MAPK8IP2*
HSA04150_MTOR_signaling_pathway	*VEGFB,RICTOR,PGF,AKT1,PRKAA1,RPS6KA3,RPS6KA6,RPS6KA1,IGF1,RPS6KB2,PIK3R1,EIF4B,MAPK3,VEGFC,AKT3,FIGF, PIK3CD,PIK3CG*
HSA05221_Acute_myeloid_ leukemia	*PIM2,NFKB2,STAT3,CCND1,FLT3,MAP2K1,CEBPA,MAP2K2,RAF1,RPS6KB2,JUP,MAPK3,TCF7,AKT3,BAD,ARAF,PIK3CD, PIK3CG,KIT*

### *NFATC3* is a commonly inactivated TSG in NHL cases and is significantly upregulated in cases with LOH of *PTPRJ*

**Figure 4:**
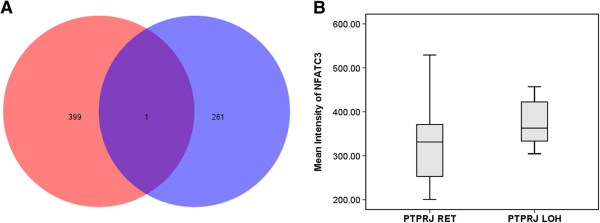
**Identification of genes located within the common LOH regions in NHL cases whose expression is affected by the LOH status of** ***PTPRJ***. **A)** Comparison of the differentially expressed genes between NHL cases with retention and LOH of *PTPRJ* (*red circle*) and genes located within the common LOH regions in NHL cases (*blue circle*) discovered that *NFATC3* gene in addition to being inactivated in NHL subtypes, **B)** is upregulated in cases with LOH of *PTPRJ. NFATC3* mean fluorescence intensity values were obtained from an Illumina Sentrix Human-6 (v2.0) Expression Beadchip [7].

In order to identify genes, previously found to be inactivated in our high resolution LOH study [8], and that are differentially expressed in the context of LOH of *PTPRJ*, we compared 262 candidate TSGs located within LOH regions against the 400 genes with differential expression between cases with retention and LOH of *PTPRJ*. This approach identified only the *NFATC3* (nuclear factor of activated T-cells, cytoplasmic, calcineurin-dependent 3) gene, located at 16q22, to be commonly inactivated and differentially expressed in NHL cases with LOH of *PTPRJ* (Figure 4). Comparison of the mean fluorescence intensity values for *NFATC3* between NHL cases with retention and LOH of *PTPRJ* revealed that *NFATC3* was significantly upregulated in cases with LOH of *PTPRJ* (325 vs. 375; *P* = 0.042)*.* Furthermore, It is important to mention that LOH of *NFATC3* was found in 58% of NHL, 48% of FL and 67% of DLBCL cases [8].

## Discussion

The implementation of pathway analyses on candidate tumour suppressor genes (TSGs) found to be targeted by loss of heterozygosity in NHL, provided a holistic perspective of the shared cellular mechanisms underlying the lymphomagenesis of the commonest forms of NHL, FL and DLBCL. This analytical approach unmasked the pathways that are mostly altered by the genetic inactivation of TSGs potentially caused by LOH events in NHL and revealed common pathogenic mechanisms between FL and DLBCL. The analysis of candidate tumour suppressor genes, previously identified to be located within common LOH regions across FL and DLBCL cases [8], via interactome and pathway analytical approaches, identified the KEGG METABOLIC pathway (hsa-01100) and the ARGININE AND PROLINE METABOLISM pathway (hsa-00330) as the most enriched pathways in relation to these candidate TSGs. In addition, this finding suggests that the regulation of these pathways might be commonly altered in NHL. These candidate TSGs encode proteins (i.e., enzymes) that may directly or indirectly (i.e., transcriptional regulators) disrupt the activity of these pathways ,either by reduced or null expression of these genes with tumour suppressor activity or by the encoding of dysfunctional proteins, resulting from the LOH-induced inactivation of these TSGs.

Disruption of metabolic pathways such as energy production or biosynthesis of amino acids, nucleosides, etc. are one of the hallmarks in cancer, as the malignant cells demand a higher activity in energy metabolism and biosynthesis in order to sustain their constitutive and accelerated proliferation and their malignant performance [25, 26]. The purpose of this metabolic reprogramming of tumour cells is not only to increase the energy output in these cells but it is also to maximize the biosynthetic pathways of the malignant cells to turn them into more efficient and self-sustaining organisms [25–27]. For instance, reprogramming of the glutamine-proline-arginine metabolic circuit has been associated with cancer and found to be regulated by TP53 and MYC, in which proline acts as the regulatory axis of this circuit [27, 28]. Therefore, the significant enrichment of candidate TSGs in the metabolic pathways related to arginine and proline metabolism in our samples is evidence that this reprogramming of energetic and biosynthetic pathways is important in NHL tumour cells. However, further functional studies are required to corroborate the effect of LOH events targeting these TSGs in the function of this metabolic pathway and its role in the tumourigenesis of FL and DLBCL.

Using an annotated network database enriched with protein-protein interactions and associations, we were able to visualize the interactome of PTPRJ in relation to candidate TSGs located within LOH regions. This analysis determined that PTPRJ does not interact directly with any other possible LOH genes; however, this TSG indirectly interacts with 22 possible inactivated TSGs by LOH in NHL cases. This finding suggests that the malignant phenotype of tumoural B-cells is orchestrated by an uncontrolled activation of oncogenic signaling pathways, which may be caused by the inactivation of the dephosphorylation activity of PTPRJ together with the inactivation of other TSGs that might regulate direct targets of PTPRJ within these pathways. For instance, PTPRJ interacts directly with GRB2, a pivotal protein in signal transduction that activates the RAS-MAPK pathway, which is inactivated by the dephosphorylation activity of PTPRJ. GRB2 was also found to interact directly with B2M in an unclear manner [29]. Interestingly, *B2M* was found to be inactivated in NHLs and its inactivation has been associated as a mechanism to evade the immune surveillance in DLBCL and other types of cancer [30, 31]. In addition, this finding also indicates that lymphomagenesis could be orchestrated by a network of TSGs that are inactivated in malignant B-cells to block tumour suppressor signalling and constitutively maintain pathogenic signals.

*PTPN11*, highly targeted by LOH events in NHL, was also identified in the PTPRJ interactome. *PTPN11* encodes a protein tyrosine phosphatase (PTP) with two SH2 domains which acts as an intracellular signalling transducer of growth factors and cytokines receptors by regulation of the MAPK pathway [32]. Furthermore, it has been demonstrated that this PTP plays a crucial role in hematopoiesis and that mutations in *PTPN11* block the T-cell and B-cell development [33]. Mutations in the *PTPN11* gene have been detected in low frequencies in leukemia cases; however, alterations in this gene predispose patients with Noolan syndrome to several types of leukemia (mainly juvenile myelomonocytic leukemia JMML) and other types of cancer. [34–36]. Interestingly, PTPN11 and PTPRJ share around 24 gene interactions, which enriched important pathways in cancer such as ERBB signalling pathway, cytokine-cytokine interactions pathway, JAK/STAT signalling pathway and others. Thus, this finding indicates that the inactivation of these two PTPs may be a key factor in the malignant B-cell development of these NHL subtypes, as double inactivation of *PTPRJ* and *PTPN11* may induce a constitutive activation of oncogenic pathways regulated by these PTPs.

Additionally, the gene *NFATC3* was identified as being significantly upregulated in NHL cases with LOH of *PTPRJ*. Interestingly, LOH of *NFATC3* was identified to be very common across FL and DLBCL cases and copy-neutral events were found to be the driving cause of LOH of *NFATC3* in these cases, based on the previously described high resolution LOH approach in these NHL cases [8]. NFATC3 is a Ca^++^ -dependent protein that regulates T-cell activation and migration, proliferation and angiogenesis by regulation of VEGF [37]. Moreover, the abrogation of NFATC3 expression in a murine model suggested the implication of this TSG in T-cell lymphomagenesis; whereas, its implication in B-cell lymphomas has not been studied [38]. In addition, NFATC3 was hypothesized as being part of a mechanism whereby intratumoural CD4^+^CD25^+^ T-cells (T_reg_ cells) interact with activated CD4^+^ T-cells to suppress the anti-tumour activity of infiltrated CD4^+^ T-cells in B-cell NHL tumours and thus, induce immune tolerance to these tumours [39]. Interestingly, these T_reg_ cells also suppress the cytotoxic activity of CD8^+^ T-cells [40]. These findings suggest that B-cell tumours may escape the immune surveillance through inactivation of *B2M*, the malignant B-cell-mediated recruitment of T_reg_ cells and regulation of infiltrating CD4+, possibly through NFATC3, and CD8+ T-cells. Further studies are required to determine the role of *NFATC3* and its inactivation in B-cell lymphomagenesis, the copy-neutral events driving LOH of NFATC3 and its relation with the inactivation of *PTPRJ,* which is known to inhibit the TCR-mediated T-cell activation by dephosphorylation of LAT and PLCG1 pathways [14, 41].

Although gene expression data was used to infer pathways of a protein tyrosine phosphatase, which lacks a DNA-binding regulatory motif, we were able to identify genes with different patterns of expression between cases with retention and LOH of *PTPRJ*, which suggests that the inactivation of *PTPRJ* might affect pathways whose ultimate goals are to switch on/off transcription factors and as a result of this, to induce an aberrant expression of these genes. In addition, using a Gene Set Enrichment Analysis (GSEA) of the differentially expressed genes between cases with LOH and retention; several pathways related to cancer and lymphocyte function were identified to be significantly upregulated in cases with LOH. This upregulation may be due to an indirect effect of the LOH of *PTPRJ*, which may cause a constitutive activation of pathways and may result in an abnormal expression of genes downstream of these pathways. Nonetheless, further functional studies (i.e., immunoblot) are required to validate these results and confirm the effect of LOH of *PTPRJ* in the expression of these oncogenic pathways.

For instance, the VEGF signalling pathway was found as the most significant upregulated pathway in cases with LOH of PTPRJ. This finding was expected as PTPRJ dephosphorylates VEGFR-2 and inhibits the VEGF-mediated cell proliferation, migration, angiogenesis and anti-apoptosis signalling [16]. Malignant activation of the VEGF signalling pathway has been widely implicated in the tumour growth and lymphangiogenesis of NHLs and the expression of VEGF has been considered as a poor prognostic factor in these hematological malignancies and has been implicated in transformation of FL to DLBCL [42–44]. Furthermore, the ERBB and MAPK signalling pathway were also found to be upregulated in cases with LOH. These oncogenic pathways have been previously described to be dephosphorylated by PTPRJ [17, 45, 46]. Likewise, as PTPRJ also affects changes in Ca^++^ concentration [19, 47], it was expected that inactivation of PTPRJ upregulates Ca^++^ signalling pathways. Unexpectedly, signalling mediated by the Fc epsilon receptor I was found to be also upregulated in LOH cases; however, despite that this receptor for IgE is expressed in early stages of B-cells [48], its relation with PTPRJ is unclear.

On the other hand, the most enriched pathways in cases with retention of PTPRJ were DNA polymerase, TP53 signaling and proteasome pathways. It is likely that the upregulation of these pathways in cells with functional PTPRJ are the consequence of the activation of tumour suppressor activities, such as controlling cell proliferation and proapoptotic signalling through dephosphorylation of VEGFR, MAPK and PI3K pathways [16–18]. The upregulation of proteasome pathway in cases with retention of PTPRJ correlates with a previous study, which found that a rat homolog of PTPRJ controlled the proteasome-mediated degradation rate of its regulator (p27^Ki^) by activation of MAPK pathway [49]. Furthermore, an analysis using freely available gene expression profiling data from FL and DLBCL cases expressing high and low levels of PTPRJ [50, 51], determined a low correlation of genes affected by *PTPRJ* transcript abundance between FL and DLBCL cases. This discrepancy may indicate differences in the LOH-driving events targeting this TSG in FLs and DLBCLs, which is supported by the significant downregulation of PTPRJ in FL cases, but not in DLBCL with LOH [8].

## Conclusion

In conclusion, the use of pathway analytical approaches has provided more evidence supporting the presence of common pathogenic mechanisms underlying the lymphomagenesis of FL and DLBCL. Specifically, this work identified genes and pathways affected by LOH of *PTPRJ.* Furthermore, these findings suggest that PTPRJ plays a crucial role in the lymphomagenesis of FL and DLBCL as this TSG was found to induce aberrant expression of genes and PTPRJ-regulated pathways in NHL cases with LOH. Finally, it is important to highlight that the role of the most promising TSGs and pathways affected by LOH in FL and DLBCL tumourigenesis will need to be validated in further *in vitro* modeling experiments.

## Methods

### LOH pathway analyses

A total of 42 NHL cases, 21 DLBCL and 21 FL, were analysed using a high resolution LOH approach, which identified 46 common LOH regions across FL and DLBCL cases, harbouring 262 candidate tumour suppressor genes [8]. The global interactome of genes affected by LOH events in NHL cases was built inserting these 262 candidate TSGs in the VisANT v. 4.06 (Integrative Visual Analysis Tool for Biological Networks and Pathways) platform to determine the level interaction amongst them. Genes without any interaction were eliminated from the analysis. In addition, a gene set enrichment analysis (GSEA) was performed on these 262 candidate TSGs, in order to investigate candidate TSGs with collections of curated gene sets, such as canonical pathways, Biocarta, KEGG, and Reactome, using the molecular signature database (MsigDB, v3.1). The significance level for this GSEA analysis was set at α < 0.05. Both analyses were based on the assumption that all the genes located within the inferred LOH regions, previously described [8], were being targeted by LOH events in the cohort of NHL cases.

### Interactome of PTPRJ

In order to extend on recently published findings implicating *PTPRJ* as a novel TSG in the lymphomagenesis of FL and DLBCL [8] and to understand the unclear role of PTPRJ in B-cell tumourigenesis, we used bioinformatics tools to investigate the effect of LOH of *PTPRJ* on genes and pathways. Thus, to identify candidate TSGs affected by LOH events that interact directly or indirectly with PTPRJ and play a role together with PTPRJ, within common cellular networks, we extracted and analyzed the interactome of PTPRJ from the global interactome enriched with the 262 candidate TSGs [8], using the VisANT v. 4.06 platform (Integrative Visual Analysis Tool for Biological Networks and Pathways). In addition, the interactome of PTPRJ was enriched with log2-transformed data from an Illumina Sentrix Human-6 (v2.0) Expression Beadchip from NHL cases (19 FLs and 20 DLBCLs) with a known LOH status (15 RET and 14 LOH) [7, 8].

### Selection of differentially expressed genes

To determine differentially expressed genes between cases with retention and LOH of *PTPRJ*, we used our previously described linear gene expression profiling (GEP) data from cases (19 FLs and 20 DLBCLs) with a known LOH status (15 RET and 14 LOH) [7, 8]. The ComparativeMarkerSelection module of the Genepattern platform [52] was employed for this selection and the analysis was carried out using the default options (2-sided T-Test and 10000 permutations). Results from this analysis were visualized using the ComparativeMarkerSelectionViewer module. A total of 400 (200 per status) differentially expressed genes were selected by the ExtractorComparativeMarkerResults module based on their score. Expression patterns were visualized using the HeatMapViewer module. A Venn diagram was used to identify common genes found to be differentially expressed from the comparison of cases with RET and LOH of *PTPRJ* and the 262 candidate TSGs identified within common LOH regions in NHL cases [8]. Comparison of the expression levels of *NFATC3* (nuclear factor of activated T-cells, cytoplasmic 3) between cases with retention and LOH of *PTPRJ* was carried out using an independent t-test (α < 0.05).

### Gene set enrichment analysis (GSEA)

The identification of gene sets that were upregulated in cases with RET and LOH of *PTPRJ* was performed using the above mentioned GEP data from NHL cases with a known LOH status (15 RET and 14 LOH) for PTPRJ in the GSEA v 2.0 software. The collection of curated pathways from the KEGG database and the default settings (without collapsing GEP data) were used for this analysis. Significance level was set at an α < 0.05.

## Electronic supplementary material

Additional file 1: **Classification of LOH genes into curated gene families.** (DOC 32 KB)

Additional file 2: **List of gene sets with chemical and genetic perturbations (CGP) enriched with genes within LOH regions.** (DOC 46 KB)

Additional file 3: **Gene sets upregulated in cases with retention (RET) of** ***PTPRJ.*** (DOC 128 KB)

Additional file 4: **Gene sets upregulated in cases with LOH of** ***PTPRJ.*** (DOC 151 KB)
