# Supplementary material for: In silico analyses reveal common cellular pathways affected by loss of heterozygosity (LOH) events in the lymphomagenesis of Non-Hodgkin’s lymphoma (NHL)
Source: BMC Genomics. 2014 May 21;15(1):390. doi: 10.1186/1471-2164-15-390 (PMC4041994; doi:10.1186/1471-2164-15-390)
Supplement: Supplementary file 1 — Additional file 1: Classification of LOH genes into curated gene families. (DOC 32 KB) [file 12864_2014_6081_MOESM1_ESM.doc]

**Additional file 1.** Classification of LOH genes into curated gene families

| **Gene Family** | **# genes** | **Genes** |
| --- | --- | --- |
| Cytokines and growth factors | 6 | *AGRP, CKLF, CMTM4,* ***FASLG****, GDF5, MANF* |
| Transcription factors | 20 | *ADPGK, ATXN2, CEBPD, CUX2, DMRTA2,* ***EP300****, MNAT1,* ***NFATC3****, NR2E3, PATZ1, RNF41, SCAND1, SIX1, SIX6, TEF, ZNF165, ZNF187, ZNF192, ZNF267, ZNF271* |
| Homeodomain | 3 | *CUX2, SIX1, SIX6* |
| Cell differentiation markers | 4 | *CXCR4,* ***FASLG****, ITGB3,* ***PTPRJ*** |
| Protein kinases | 8 | *ERBB3, LIMK2, MAPK6, MAPKAPK5, MYLK3, PRKDC, TTBK2, YSK4* |
| Translocated cancer genes | 7 | *ACSL6, CBFB,* ***EP300****, EPS15, GPHN, HOOK3, PATZ1* |
| Oncogenes | 7 | *ACSL6, CBFB, EPS15, GPHN, HOOK3, PATZ1,* ***PTPN11*** |
| Tumor suppressor genes | 2 | *CDKN2C,* ***EP300*** |
