# Supplementary material for: In silico analyses reveal common cellular pathways affected by loss of heterozygosity (LOH) events in the lymphomagenesis of Non-Hodgkin’s lymphoma (NHL)
Source: BMC Genomics. 2014 May 21;15(1):390. doi: 10.1186/1471-2164-15-390 (PMC4041994; doi:10.1186/1471-2164-15-390)
Supplement: Supplementary file 2 — Additional file 2: List of gene sets with chemical and genetic perturbations (CGP) enriched with genes within LOH regions. (DOC 46 KB) [file 12864_2014_6081_MOESM2_ESM.doc]

**Additional file 2. List of gene sets with chemical and genetic perturbations (CGP) enriched with genes within LOH regions**

| **Gene Set Name** | **K** | **Description** | **k** | **k/K** | ***P* value** |
| --- | --- | --- | --- | --- | --- |
| **NIKOLSKY_BREAST_CANCER_20Q11_AMPLICON** | 31 | Genes within amplicon 20q11 identified in a copy number alterations study of 191 breast tumor samples. | 8 | 0.2581 | 8.08 x10^-10^ |
| **ROYLANCE_BREAST_CANCER_16Q_COPY_NUMBER_DN** | 26 | Genes in discrete regions of loss within 16q region detected in individual invasive breast cancer tumors. | 6 | 0.2308 | 2.36 x10^-7^ |
| **MARSON_BOUND_BY_FOXP3_STIMULATED** | 1022 | Genes with promoters bound by FOXP3 [GeneID=50943] in hybridoma cells stimulated by PMA [PubChem=4792] and ionomycin [PubChem=3733]. | 28 | 0.0274 | 3.14 x10^-6^ |
| **DIAZ_CHRONIC_MEYLOGENOUS_LEUKEMIA_UP** | 1382 | Genes up-regulated in CD34+ [GeneID=947] cells isolated from bone marrow of CML (chronic myelogenous leukemia) patients, compared to those from normal donors. | 31 | 0.0224 | 4.76 x10^-5^ |
| **GRAESSMANN_RESPONSE_TO_MC_AND_DOXORUBICIN_UP** | 612 | Genes up-regulated in ME-A cells (breast cancer, sensitive to apoptotic stimuli) exposed to doxorubicin [PubChem=31703] in the presence of medium concentrate (MC) from ME-C cells (breast cancer, resistant to apoptotic stimuli). | 17 | 0.0278 | 2.56 x10^-4^ |
| **ZHAN_MULTIPLE_MYELOMA_SUBGROUPS** | 30 | Top genes up-regulated in MM4 vs MM1 subgroup of multiple myeloma samples. | 4 | 0.1333 | 2.58 x10^-4^ |
| **YOSHIMURA_MAPK8_TARGETS_DN** | 366 | Genes down-regulated in vascular smooth muscle cells (VSMC) by MAPK8 (JNK1) [GeneID=5599]. | 12 | 0.0328 | 5.00 x10^-4^ |
| **FIRESTEIN_PROLIFERATION** | 175 | Genes required for proliferation of DLD-1 cell (colon cancer with APC [GeneID=324] deletions), based on shRNA screen. | 8 | 0.0457 | 5.25 x10^-4^ |
| **PUJANA_BREAST_CANCER_LIT_INT_NETWORK** | 101 | Genes constituting the LIT-Int network of proteins interacting with breast cancer reference proteins BRCA1, BRCA2, ATM, and CHEK2 [GeneID=672;675;472;11200]. | 6 | 0.0594 | 6.78 x10^-4^ |
| **ZHENG_RESPONSE_TO_ARSENITE_DN** | 18 | Down-regulated in HEK293 cells (kidney epithelium) by treatment with sodium arsenite [PubChem=26435]. | 3 | 0.1667 | 8.20 x10^-4^ |
| **SENESE_HDAC2_TARGETS_UP** | 114 | Genes up-regulated in U2OS cells (osteosarcoma) upon knockdown of HDAC2 [GeneID=3066] by RNAi. | 6 | 0.0526 | 1.28 x10^-3^ |
| **FUJII_YBX1_TARGETS_DN** | 202 | Genes down-regulated in MCF-7 cells (breast cancer) after knockdown of YBX1 [GeneID=4904] by RNAi. | 8 | 0.0396 | 1.33 x10^-3^ |
| **GAUSSMANN_MLL_AF4_FUSION_TARGETS_E_DN** | 22 | Down-regulated genes from the set E (Fig. 5a): specific signature shared by cells expressing either MLL-AF4 [GeneID=4297;4299] or AF4-MLL fusion proteins alone, | 3 | 0.1364 | 1.50 x10^-3^ |
| **KUNINGER_IGF1_VS_PDGFB_TARGETS_UP** | 82 | Genes up-regulated in C2AS12 cells (myoblast) by IGF1 [GeneID=3479] vs PDGFB [GeneID=5155]. | 5 | 0.061 | 1.70 x10^-3^ |

* k/K is the ratio between the # of genes in overlap (k) and the # of genes in gene set (K).
