# Supplementary material for: In silico analyses reveal common cellular pathways affected by loss of heterozygosity (LOH) events in the lymphomagenesis of Non-Hodgkin’s lymphoma (NHL)
Source: BMC Genomics. 2014 May 21;15(1):390. doi: 10.1186/1471-2164-15-390 (PMC4041994; doi:10.1186/1471-2164-15-390)
Supplement: Supplementary file 3 — Additional file 3: Gene sets upregulated in cases with retention (RET) of PTPRJ. (DOC 128 KB) [file 12864_2014_6081_MOESM3_ESM.doc]

***Additional file 3.*** *Gene sets upregulated in cases with retention (RET) of PTPRJ*

| Gene Set Name | Size | ES | NES | NOM p-val | FDR q-val | FWER p-val | |  |
| --- | --- | --- | --- | --- | --- | --- | --- | --- |
| HSA03030_DNA_POLYMERASE | 23 | 0.58100396 | 1.579797 | 0.04590818 | 1 | 0.634 |  | |
| HSA04115_P53_SIGNALING_PATHWAY | 67 | 0.39780596 | 1.427746 | 0.08007449 | 1 | 0.886 |  | |
| HSA03050_PROTEASOME | 22 | 0.5875306 | 1.42035 | 0.15830116 | 1 | 0.894 |  | |
| HSA00970_AMINOACYL_TRNA_BIOSYNTHESIS | 36 | 0.51731735 | 1.392424 | 0.17382812 | 1 | 0.923 |  | |
| HSA04110_CELL_CYCLE | 113 | 0.41090462 | 1.387758 | 0.17692308 | 1 | 0.926 |  | |
| HSA00271_METHIONINE_METABOLISM | 17 | 0.52018476 | 1.325884 | 0.19702603 | 1 | 0.968 |  | |
| HSA00670_ONE_CARBON_POOL_BY_FOLATE | 16 | 0.52224094 | 1.287694 | 0.21062993 | 1 | 0.985 |  | |
| HSA00790_FOLATE_BIOSYNTHESIS | 42 | 0.370707 | 1.265893 | 0.21641791 | 1 | 0.991 |  | |
| HSA00010_GLYCOLYSIS_AND_GLUCONEOGENESIS | 63 | 0.3417722 | 1.259978 | 0.15425532 | 1 | 0.991 |  | |
| HSA00052_GALACTOSE_METABOLISM | 32 | 0.3959104 | 1.206927 | 0.26119402 | 1 | 0.997 |  | |
| HSA00620_PYRUVATE_METABOLISM | 42 | 0.35203496 | 1.182028 | 0.24907748 | 1 | 0.999 |  | |
| HSA00310_LYSINE_DEGRADATION | 47 | 0.34018207 | 1.17216 | 0.23920864 | 1 | 0.999 |  | |
| HSA00230_PURINE_METABOLISM | 142 | 0.28853348 | 1.157259 | 0.27436823 | 1 | 0.999 |  | |
| HSA00500_STARCH_AND_SUCROSE_METABOLISM | 84 | 0.29027018 | 1.156868 | 0.2437276 | 1 | 0.999 |  | |
| HSA00770_PANTOTHENATE_AND_COA_BIOSYNTHESIS | 16 | 0.40341887 | 1.14969 | 0.30570903 | 1 | 0.999 |  | |
| HSA00860_PORPHYRIN_AND_CHLOROPHYLL_METABOLISM | 41 | 0.35514876 | 1.146116 | 0.27592593 | 1 | 0.999 |  | |
| HSA00530_AMINOSUGARS_METABOLISM | 29 | 0.3831149 | 1.139467 | 0.3251418 | 1 | 0.999 |  | |
| HSA00240_PYRIMIDINE_METABOLISM | 85 | 0.34292036 | 1.138806 | 0.34545454 | 1 | 0.999 |  | |
| HSA00440_AMINOPHOSPHONATE_METABOLISM | 16 | 0.39853242 | 1.133307 | 0.30812854 | 1 | 0.999 |  | |
| HSA01510_NEURODEGENERATIVE_DISEASES | 38 | 0.3058341 | 1.084853 | 0.33592233 | 1 | 1 |  | |
| HSA00640_PROPANOATE_METABOLISM | 34 | 0.34461343 | 1.080232 | 0.35809523 | 1 | 1 |  | |
| HSA05010_ALZHEIMERS_DISEASE | 28 | 0.34414318 | 1.064971 | 0.37205082 | 1 | 1 |  | |
| HSA00564_GLYCEROPHOSPHOLIPID_METABOLISM | 68 | 0.26599726 | 1.059467 | 0.36476868 | 1 | 1 |  | |
| HSA00280_VALINE_LEUCINE_AND_ISOLEUCINE_DEGRADATION | 43 | 0.33501312 | 1.05633 | 0.40118578 | 1 | 1 |  | |
| HSA00360_PHENYLALANINE_METABOLISM | 30 | 0.3354395 | 1.044786 | 0.38817006 | 1 | 1 |  | |
| HSA04530_TIGHT_JUNCTION | 135 | 0.23285607 | 1.041278 | 0.39716312 | 1 | 1 |  | |
| HSA05050_DENTATORUBROPALLIDOLUYSIAN_ATROPHY | 15 | 0.34242612 | 1.040001 | 0.4016227 | 0.979713 | 1 |  | |
| HSA02010_ABC_TRANSPORTERS_GENERAL | 44 | 0.2883836 | 1.035598 | 0.39855072 | 0.95849 | 1 |  | |
| HSA01430_CELL_COMMUNICATION | 135 | 0.3243238 | 1.029354 | 0.4117647 | 0.943631 | 1 |  | |
| HSA05130_PATHOGENIC_ESCHERICHIA_COLI_INFECTION | 51 | 0.26257157 | 1.023512 | 0.42039356 | 0.929616 | 1 |  | |
| HSA05131_PATHOGENIC_ESCHERICHIA_COLI_INFECTION | 51 | 0.26257157 | 1.023512 | 0.42039356 | 0.899628 | 1 |  | |
| HSA00330_ARGININE_AND_PROLINE_METABOLISM | 35 | 0.31291652 | 0.98827 | 0.47985348 | 0.973659 | 1 |  | |
| HSA00960_ALKALOID_BIOSYNTHESIS_II | 21 | 0.32548502 | 0.985987 | 0.48758864 | 0.951106 | 1 |  | |
| HSA04140_REGULATION_OF_AUTOPHAGY | 30 | 0.283958 | 0.974448 | 0.48785424 | 0.952882 | 1 |  | |
| HSA04620_TOLL_LIKE_RECEPTOR_SIGNALING_PATHWAY | 102 | 0.23564987 | 0.954974 | 0.48983365 | 0.982048 | 1 |  | |
| HSA00020_CITRATE_CYCLE | 29 | 0.34181237 | 0.939585 | 0.5536398 | 0.999129 | 1 |  | |
| HSA00361_GAMMA_HEXACHLOROCYCLOHEXANE_DEG | 23 | 0.2984337 | 0.936171 | 0.5344203 | 0.981778 | 1 |  | |
| HSA05030_AMYOTROPHIC_LATERAL_SCLEROSIS | 19 | 0.2856566 | 0.926231 | 0.5557554 | 0.984205 | 1 |  | |
| HSA04610_COMPLEMENT_AND_COAGULATION_CASC | 68 | 0.32377377 | 0.915163 | 0.54414415 | 0.988828 | 1 |  | |
| HSA00272_CYSTEINE_METABOLISM | 17 | 0.3153253 | 0.908121 | 0.58003765 | 0.981281 | 1 |  | |
| HSA04540_GAP_JUNCTION | 96 | 0.20414397 | 0.903936 | 0.6461825 | 0.968226 | 1 |  | |
| HSA04920_ADIPOCYTOKINE_SIGNALING_PATHWAY | 72 | 0.21892917 | 0.884911 | 0.653913 | 0.992456 | 1 |  | |
| HSA00591_LINOLEIC_ACID_METABOLISM | 31 | 0.26455265 | 0.843921 | 0.7082601 | 1 | 1 |  | |
| HSA00340_HISTIDINE_METABOLISM | 41 | 0.22879817 | 0.835604 | 0.7447183 | 1 | 1 |  | |
| HSA00710_CARBON_FIXATION | 23 | 0.3110164 | 0.829621 | 0.6332737 | 1 | 1 |  | |
| HSA04330_NOTCH_SIGNALING_PATHWAY | 46 | 0.21157098 | 0.818458 | 0.7479839 | 1 | 1 |  | |
| HSA00642_ETHYLBENZENE_DEGRADATION | 15 | 0.28682613 | 0.811495 | 0.72168905 | 1 | 1 |  | |
| HSA04614_RENIN_ANGIOTENSIN_SYSTEM | 17 | 0.2772285 | 0.798317 | 0.72477067 | 1 | 1 |  | |
| HSA00252_ALANINE_AND_ASPARTATE_METABOLISM | 33 | 0.2575419 | 0.795869 | 0.69758815 | 1 | 1 |  | |
| HSA05020_PARKINSONS_DISEASE | 15 | 0.26881465 | 0.781973 | 0.7581574 | 1 | 1 |  | |
| HSA00251_GLUTAMATE_METABOLISM | 30 | 0.25869694 | 0.781758 | 0.76079136 | 1 | 1 |  | |
| HSA00410_BETA_ALANINE_METABOLISM | 25 | 0.24075949 | 0.758147 | 0.7798507 | 1 | 1 |  | |
| HSA00450_SELENOAMINO_ACID_METABOLISM | 27 | 0.26416045 | 0.752652 | 0.74248123 | 1 | 1 |  | |
| HSA00930_CAPROLACTAM_DEGRADATION | 15 | 0.25271437 | 0.739833 | 0.8123791 | 1 | 1 |  | |
| HSA00260_GLYCINE_SERINE_AND_THREONINE_MET | 45 | 0.20200641 | 0.709588 | 0.90053284 | 1 | 1 |  | |
| HSA00910_NITROGEN_METABOLISM | 24 | 0.2336117 | 0.701911 | 0.8378378 | 1 | 1 |  | |
| HSA03022_BASAL_TRANSCRIPTION_FACTORS | 32 | 0.24514848 | 0.69576 | 0.791423 | 1 | 1 |  | |
| HSA03320_PPAR_SIGNALING_PATHWAY | 68 | 0.20135547 | 0.682591 | 0.8447972 | 1 | 1 |  | |
| HSA05216_THYROID_CANCER | 29 | 0.20026973 | 0.669731 | 0.8833333 | 1 | 1 |  | |
| HSA00040_PENTOSE_AND_GLUCURONATE_INTERCONV | 25 | 0.2361259 | 0.668206 | 0.8925926 | 1 | 1 |  | |
| HSA00903_LIMONENE_AND_PINENE_DEGRADATION | 29 | 0.20306139 | 0.663278 | 0.91566265 | 1 | 1 |  | |
| HSA00632_BENZOATE_DEGRADATION_VIA_COA_LIG | 27 | 0.19994035 | 0.657941 | 0.92263055 | 1 | 1 |  | |
| HSA00534_HEPARAN_SULFATE_BIOSYNTHESIS | 19 | 0.22396442 | 0.652286 | 0.8994614 | 1 | 1 |  | |
| HSA00071_FATTY_ACID_METABOLISM | 45 | 0.18553552 | 0.628304 | 0.9383698 | 1 | 1 |  | |
| HSA00563_GLYCOSYLPHOSPHATIDYLINOSITOL_ANCHOR | 23 | 0.18857758 | 0.559729 | 0.94538605 | 1 | 1 |  | |
| HSA00511_N_GLYCAN_DEGRADATION | 16 | 0.18425316 | 0.492864 | 0.9723757 | 1 | 1 |  | |
| HSA00510_N_GLYCAN_BIOSYNTHESIS | 41 | 0.16054136 | 0.474464 | 0.97333336 | 1 | 1 |  | |
| HSA01032_GLYCAN_STRUCTURES_DEGRADATION | 30 | 0.16692609 | 0.464651 | 0.9706458 | 1 | 1 |  | |
| HSA00190_OXIDATIVE_PHOSPHORYLATION | 113 | 0.1303609 | 0.423496 | 0.99811673 | 1 | 1 |  | |
| HSA03010_RIBOSOME | 80 | 0.13241345 | 0.415878 | 0.96511626 | 0.997159 | 1 |  | |
|  | | | | | | |  | |
