# Supplementary material for: In silico analyses reveal common cellular pathways affected by loss of heterozygosity (LOH) events in the lymphomagenesis of Non-Hodgkin’s lymphoma (NHL)
Source: BMC Genomics. 2014 May 21;15(1):390. doi: 10.1186/1471-2164-15-390 (PMC4041994; doi:10.1186/1471-2164-15-390)
Supplement: Supplementary file 4 — Additional file 4: Gene sets upregulated in cases with LOH of PTPRJ. (DOC 151 KB) [file 12864_2014_6081_MOESM4_ESM.doc]

**Additional file 4.** Gene sets upregulated in cases with LOH of *PTPRJ*

| Name | Size | ES | NES | NOM p-val | FDR q-val | FWER p-val |
| --- | --- | --- | --- | --- | --- | --- |
| HSA04370_VEGF_SIGNALING_PATHWAY | 70 | -0.362535 | -1.5681 | 0.002109705 | 1 | 0.613 |
| HSA05218_MELANOMA | 71 | -0.364067 | -1.5187 | 0.02586207 | 1 | 0.72 |
| HSA04012_ERBB_SIGNALING_PATHWAY | 87 | -0.334774 | -1.4485 | 0.033264033 | 1 | 0.82 |
| HSA05223_NON_SMALL_CELL_LUNG_CANCER | 54 | -0.37297 | -1.4431 | 0.069327734 | 1 | 0.823 |
| HSA04020_CALCIUM_SIGNALING_PATHWAY | 172 | -0.315852 | -1.419 | 0.020361992 | 1 | 0.851 |
| HSA04930_TYPE_II_DIABETES_MELLITUS | 44 | -0.361928 | -1.3991 | 0.060737528 | 1 | 0.872 |
| HSA05214_GLIOMA | 64 | -0.356184 | -1.3773 | 0.10559006 | 1 | 0.886 |
| HSA04664_FC_EPSILON_RI_SIGNALING_PATHWAY | 75 | -0.311534 | -1.3198 | 0.086283185 | 1 | 0.934 |
| HSA05211_RENAL_CELL_CARCINOMA | 69 | -0.324027 | -1.3117 | 0.11764706 | 1 | 0.939 |
| HSA04010_MAPK_SIGNALING_PATHWAY | 254 | -0.267518 | -1.3065 | 0.06575964 | 1 | 0.944 |
| HSA04150_MTOR_SIGNALING_PATHWAY | 48 | -0.325553 | -1.2993 | 0.12371134 | 1 | 0.947 |
| HSA05221_ACUTE_MYELOID_LEUKEMIA | 53 | -0.339594 | -1.2899 | 0.14699793 | 0.9654402 | 0.951 |
| HSA05212_PANCREATIC_CANCER | 73 | -0.309422 | -1.2849 | 0.11011236 | 0.91078556 | 0.952 |
| HSA00604_GLYCOSPHINGOLIPID_BIOSYNTHESIS | 16 | -0.44628 | -1.2534 | 0.20762712 | 0.97297513 | 0.969 |
| HSA00592_ALPHA_LINOLENIC_ACID_MET | 15 | -0.427229 | -1.2494 | 0.16783217 | 0.9236814 | 0.971 |
| HSA00760_NICOTINATE_AND_NICOTINAMIDE_MET | 23 | -0.357448 | -1.2291 | 0.19406393 | 0.9452058 | 0.976 |
| HSA05213_ENDOMETRIAL_CANCER | 52 | -0.315122 | -1.2237 | 0.17959183 | 0.91067487 | 0.977 |
| HSA04910_INSULIN_SIGNALING_PATHWAY | 135 | -0.2575 | -1.2019 | 0.14220184 | 0.94357795 | 0.982 |
| HSA04640_HEMATOPOIETIC_CELL_LINEAGE | 86 | -0.318547 | -1.2003 | 0.22717622 | 0.9015147 | 0.984 |
| HSA04730_LONG_TERM_DEPRESSION | 75 | -0.285942 | -1.1902 | 0.2 | 0.8910197 | 0.985 |
| HSA04070_PHOSPHATIDYLINOSITOL_SIGNALING | 74 | -0.297112 | -1.189 | 0.19628099 | 0.8521317 | 0.985 |
| HSA05110_CHOLERA_INFECTION | 41 | -0.309836 | -1.1866 | 0.23092784 | 0.82107395 | 0.985 |
| HSA04310_WNT_SIGNALING_PATHWAY | 147 | -0.260268 | -1.182 | 0.18722467 | 0.8006458 | 0.986 |
| HSA04810_REGULATION_OF_ACTIN_CYTO | 203 | -0.256639 | -1.1819 | 0.20169851 | 0.76748985 | 0.986 |
| HSA04940_TYPE_I_DIABETES_MELLITUS | 44 | -0.368443 | -1.1795 | 0.25056434 | 0.7431212 | 0.987 |
| HSA04916_MELANOGENESIS | 101 | -0.271574 | -1.1631 | 0.20495495 | 0.76371986 | 0.991 |
| HSA04720_LONG_TERM_POTENTIATION | 68 | -0.285554 | -1.1568 | 0.25604838 | 0.7519065 | 0.991 |
| HSA04660_T_CELL_RECEPTOR_SIGNALING | 93 | -0.313458 | -1.1565 | 0.29813665 | 0.7260075 | 0.991 |
| HSA05210_COLORECTAL_CANCER | 84 | -0.276968 | -1.1433 | 0.27672955 | 0.7364345 | 0.992 |
| HSA04950_MATURITY_ONSET_DIABETES_OF | 24 | -0.358109 | -1.1403 | 0.29166666 | 0.7195197 | 0.992 |
| HSA00562_INOSITOL_PHOSPHATE_MET | 47 | -0.305614 | -1.1279 | 0.27122152 | 0.72960216 | 0.992 |
| HSA00561_GLYCEROLIPID_MET | 57 | -0.288871 | -1.1187 | 0.3026316 | 0.731614 | 0.993 |
| HSA05219_BLADDER_CANCER | 42 | -0.301318 | -1.1149 | 0.31681034 | 0.7189331 | 0.995 |
| HSA04080_NEUROACTIVE_LIGAND_RECEPTOR_ | 249 | -0.243692 | -1.1071 | 0.26303318 | 0.7183839 | 0.995 |
| HSA04670_LEUKOCYTE_TRANSENDOTHELIAL_MIG | 113 | -0.273311 | -1.105 | 0.3125 | 0.70326686 | 0.995 |
| HSA04360_AXON_GUIDANCE | 127 | -0.271317 | -1.0972 | 0.30607966 | 0.70304585 | 0.996 |
| HSA05215_PROSTATE_CANCER | 87 | -0.252414 | -1.0803 | 0.34429824 | 0.72807914 | 0.998 |
| HSA04612_ANTIGEN_PROCESSING_AND_PTION | 80 | -0.279133 | -1.0616 | 0.38510638 | 0.7579056 | 0.999 |
| HSA04320_DORSO_VENTRAL_AXIS_FORMATION | 28 | -0.306807 | -1.0441 | 0.37044534 | 0.7829937 | 0.999 |
| HSA00512_O_GLYCAN_BIOSYNTHESIS | 31 | -0.277969 | -1.0044 | 0.4517647 | 0.87102646 | 1 |
| HSA04210_APOPTOSIS | 83 | -0.234212 | -0.9957 | 0.44871795 | 0.87402195 | 1 |
| HSA00740_RIBOFLAVIN_METABOLISM | 16 | -0.326997 | -0.9949 | 0.4635193 | 0.8552567 | 1 |
| HSA04510_FOCAL_ADHESION | 196 | -0.253932 | -0.9732 | 0.47095436 | 0.8963124 | 1 |
| HSA00624_1_AND_2_METHYLNAPHTHALENE_DTION | 24 | -0.289336 | -0.9711 | 0.4989429 | 0.88221586 | 1 |
| HSA04912_GNRH_SIGNALING_PATHWAY | 97 | -0.223682 | -0.9661 | 0.47629312 | 0.8756162 | 1 |
| HSA04630_JAK_STAT_SIGNALING_PATHWAY | 153 | -0.213605 | -0.9555 | 0.5086207 | 0.88395405 | 1 |
| HSA05217_BASAL_CELL_CARCINOMA | 55 | -0.251226 | -0.9527 | 0.48723406 | 0.87292904 | 1 |
| HSA04514_CELL_ADHESION_MOLECULES | 133 | -0.256364 | -0.9439 | 0.5210643 | 0.8780113 | 1 |
| HSA00533_KERATAN_SULFATE_BIOSYNTHESIS | 16 | -0.352289 | -0.9433 | 0.53814435 | 0.8620834 | 1 |
| HSA01031_GLYCAN_STRUCTURES_ | 62 | -0.240792 | -0.9383 | 0.5237069 | 0.8571206 | 1 |
| HSA04740_OLFACTORY_TRANSDUCTION | 31 | -0.252296 | -0.9358 | 0.5538462 | 0.84683186 | 1 |
| HSA04662_B_CELL_RECEPTOR_SIGNALING_P | 64 | -0.230369 | -0.9305 | 0.5678497 | 0.8429339 | 1 |
| HSA05120_EPITHELIAL_CELL_SIGNALING_IN_HPYLORI | 68 | -0.238449 | -0.9177 | 0.59598213 | 0.85807157 | 1 |
| HSA00650_BUTANOATE_METABOLISM | 45 | -0.266899 | -0.9084 | 0.58849555 | 0.8653054 | 1 |
| HSA00532_CHONDROITIN_SULFATE_B | 18 | -0.345568 | -0.9007 | 0.5483871 | 0.8683317 | 1 |
| HSA05220_CHRONIC_MYELOID_LEUKEMIA | 76 | -0.211822 | -0.8953 | 0.6241901 | 0.8647152 | 1 |
| HSA04340_HEDGEHOG_SIGNALING_P | 57 | -0.222651 | -0.8821 | 0.6530612 | 0.88130635 | 1 |
| HSA00480_GLUTATHIONE_METABOLISM | 38 | -0.258786 | -0.8736 | 0.59170306 | 0.8853026 | 1 |
| HSA00051_FRUCTOSE_AND_MANNOSE_MET | 42 | -0.253186 | -0.8663 | 0.63398695 | 0.8866721 | 1 |
| HSA04520_ADHERENS_JUNCTION | 75 | -0.223851 | -0.8567 | 0.673774 | 0.89302593 | 1 |
| HSA00120_BILE_ACID_BIOSYNTHESIS | 36 | -0.250504 | -0.8509 | 0.68329716 | 0.89173996 | 1 |
| HSA00980_METABOLISM_OF_XENOBIOTICS_BYP450 | 69 | -0.23871 | -0.8393 | 0.6797235 | 0.90153986 | 1 |
| HSA00590_ARACHIDONIC_ACID_METABOLISM | 53 | -0.235275 | -0.8357 | 0.68201756 | 0.89493257 | 1 |
| HSA04650_NATURAL_KILLER_CELL_MEDIATED_CYTO | 130 | -0.203928 | -0.8224 | 0.6828753 | 0.9095021 | 1 |
| HSA00350_TYROSINE_METABOLISM | 58 | -0.208074 | -0.8181 | 0.7731481 | 0.90457976 | 1 |
| HSA00626_NAPHTHALENE_AND_ANTHRACENE_DTION | 18 | -0.278639 | -0.7982 | 0.70434785 | 0.9311763 | 1 |
| HSA00220_UREA_CYCLE_AND_METABOLISM_OF AA | 29 | -0.245712 | -0.7849 | 0.75974023 | 0.94380075 | 1 |
| HSA04060_CYTOKINE_CYTOKINE_RECEPTOR_ | 254 | -0.184782 | -0.7693 | 0.78118163 | 0.9597743 | 1 |
| HSA01030_GLYCAN_STRUCTURES_BIOSYNTHESIS_1 | 111 | -0.193098 | -0.7667 | 0.81737196 | 0.95049465 | 1 |
| HSA05040_HUNTINGTONS_DISEASE | 30 | -0.206027 | -0.7616 | 0.83864117 | 0.9465974 | 1 |
| HSA03020_RNA_POLYMERASE | 23 | -0.290767 | -0.7573 | 0.69684213 | 0.9404806 | 1 |
| HSA00565_ETHER_LIPID_METABOLISM | 31 | -0.225711 | -0.755 | 0.8081264 | 0.9316458 | 1 |
| HSA00030_PENTOSE_PHOSPHATE_PATHWAY | 26 | -0.255523 | -0.7249 | 0.7724289 | 0.9663192 | 1 |
| HSA04742_TASTE_TRANSDUCTION | 52 | -0.186682 | -0.7211 | 0.93028843 | 0.9587595 | 1 |
| HSA00150_ANDROGEN_AND_ESTROGEN_MET | 54 | -0.189029 | -0.6913 | 0.9092827 | 0.9856895 | 1 |
| HSA05222_SMALL_CELL_LUNG_CANCER | 87 | -0.162787 | -0.6839 | 0.9267734 | 0.9823458 | 1 |
| HSA04350_TGF_BETA_SIGNALING_PATHWAY | 88 | -0.167574 | -0.6719 | 0.9562363 | 0.9830015 | 1 |
| HSA00600_SPHINGOLIPID_METABOLISM | 38 | -0.184052 | -0.6642 | 0.96511626 | 0.9787783 | 1 |
| HSA04120_UBIQUITIN_MEDIATED_PROTEOLYSIS | 38 | -0.186817 | -0.6486 | 0.90224034 | 0.9807961 | 1 |
| HSA00380_TRYPTOPHAN_METABOLISM | 59 | -0.158953 | -0.6274 | 0.97727275 | 0.9872338 | 1 |
| HSA00531_GLYCOSAMINOGLYCAN_DTION | 17 | -0.207126 | -0.5403 | 0.9419087 | 1 | 1 |
| HSA04130_SNARE_INTERACTIONS_IN_VES_TRANS | 34 | -0.130301 | -0.4987 | 1 | 1 | 1 |
| HSA00602_GLYCOSPHINGOLIPID_BIOSYNTHESIS | 21 | -0.147137 | -0.4662 | 0.99571735 | 1 | 1 |
| HSA04512_ECM_RECEPTOR_INTERACTION | 85 | -0.15249 | -0.4477 | 0.95913976 | 1 | 1 |
| HSA00100_BIOSYNTHESIS_OF_STEROIDS | 24 | -0.145305 | -0.4032 | 0.9914347 | 0.99796456 | 1 |
